# Mechanical and Histological Effects of Resorbable Blasting Media Surface Treatment on the Initial Stability of Orthodontic Mini-Implants

**DOI:** 10.1155/2016/7520959

**Published:** 2016-01-28

**Authors:** Odontuya Gansukh, Jong-Wha Jeong, Jong-Wan Kim, Jong-Ho Lee, Tae-Woo Kim

**Affiliations:** ^1^Department of Orthodontics, School of Dentistry and Dental Research Institute, Seoul National University, Seoul 03080, Republic of Korea; ^2^Department of Orthodontics, Section of Dentistry, Seoul National University Bundang Hospital, Seongnam-si 13620, Republic of Korea; ^3^Department of Oral and Maxillofacial Surgery and Oral Cancer Center, School of Dentistry and Dental Research Institute, Seoul National University Dental Hospital, Seoul 03080, Republic of Korea

## Abstract

*Introduction*. This study aimed to evaluate the effects of resorbable blasting media (RBM) treatment on early stability of orthodontic mini-implants by mechanical, histomorphometric, and histological analyses.* Methods*. Ninety-six (64 for mechanical study and 32 for histological study and histomorphometric analysis) titanium orthodontic mini-implants (OMIs) with machined (machined group) or RBM-treated (CaP) surface (RBM group) were implanted in the tibiae of 24 rabbits. Maximum initial torque (MIT) was measured during insertion, and maximum removal torque (MRT) and removal angular momentum (RAM) were measured at 2 and 4 weeks after implantation. Bone-to-implant contact (BIC) and bone area (BA) were analyzed at 4 weeks after implantation.* Results*. RBM group exhibited significantly lower MIT and significantly higher MRT and RAM at 2 weeks than machined group. No significant difference in MRT, RAM, and BIC between the two groups was noted at 4 weeks, although BA was significantly higher in RBM group than in machined group. RBM group showed little bone resorption, whereas machined group showed new bone formation after bone resorption.* Conclusions*. RBM surface treatment can provide early stability of OMIs around 2 weeks after insertion, whereas stability of machined surface OMIs may decrease in early stages because of bone resorption, although it can subsequently recover by new bone apposition.

## 1. Introduction

In recent times, mini-implants have been used in orthodontic treatment for absolute anchorage, and these can be used to apply various orthodontic forces to the teeth because of their small size [1–3]. However, orthodontic mini-implants (OMIs) with a small diameter can be easily loosened by a small removal torque [4]; furthermore, the success rate of mini-implants with a short length has been reported to be low [5].

The failure rate of small-sized OMIs may be higher than that of conventional implants [6, 7]. In particular, the initial stability may be low [8]. The success rate of OMIs is limited by their diameter and length, because they frequently need to be inserted between tooth roots [9]. The failure rate of OMIs that are conventionally inserted in the buccal alveolar bone was reported to be approximately 10%–30% [6], which was higher than that of surgical plates or palatal implants. Owing to their small diameter, OMIs could easily loosen under loading [10], and the osseointegration could support the stability of OMI [11]. The properties of an implant surface can affect osseointegration on the surface and stability of the implant [12]. Some surface characteristics of implants, including surface composition and structure, surface energy, oxide thickness, and topography, may play important roles in the formation and maintenance of bone at the implant surface [13] and may also affect its mechanical properties [14].

The surface roughness of implants appears to be the factor that maximizes new bone formation [15–17]. The surface roughness of the implant can affect cell function and matrix deposition and mineralization [18]. Bone growth into the micropores on the implant may result in mechanical interlocking between the implant and bone, leading to a stronger bone-implant interface [19]. Recently, sand blasting of dental implant surfaces with resorbable blasting media (RBM) such as hydroxyapatite or calcium phosphate particles was reported [20]. Furthermore, it was reported that RBM treatment is associated with a greater removal torque and interfacial bone contact compared with machined implants and appears to have the most benefit on early bone formation and initial implant stability [21].

This study aimed to analyze the histological and mechanical effects of RBM surface treatment on the initial stability of OMIs in comparing removal torque and histomorphometric parameters such as bone-to-implant contact (BIC) and bone area (BA). It also aimed to determine the healing processes with both implant surface types by conducting histological and fluorescence evaluations. It was hypothesized that RBM surface treatment would not significantly affect the initial stability of OMIs.

## 2. Materials and Methods

### 2.1. Implants

**Figure 1:**
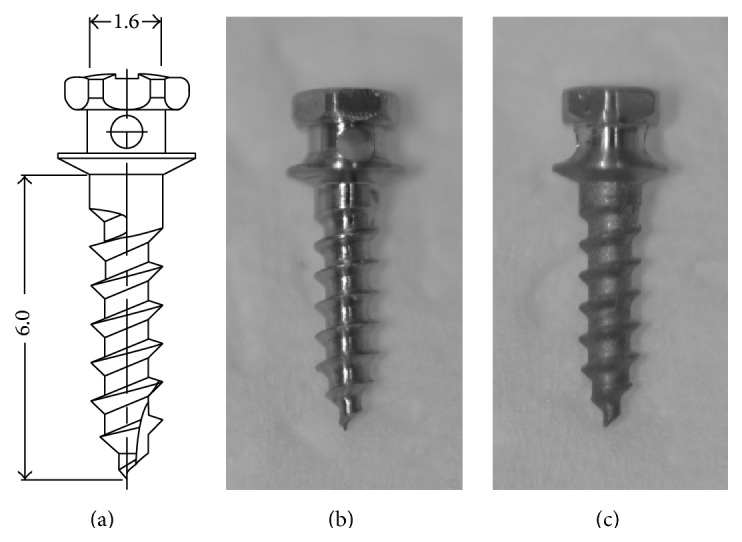
(a) Design of the mini-implant, which had a diameter of 1.6 mm and a length of 6.0 mm. (b) Machined group. (c) Resorbable blasting media (RBM) group.

Ninety-six titanium OMIs (length, 6.0 mm; diameter, 1.6 mm; Dual-Top®, Jeil Medical Corporation, Seoul, Republic of Korea), composed of Ti-6Al-4V alloy (Table 1), were used in this study. Of the 96, 64 were used for mechanical studies (32 for removal at 2 weeks and 32 for removal at 4 weeks) and 32 for specimen preparation of histological studies and histomorphometric analyses (Table 2). The surfaces of these implants were either machine-treated (48 OMIs, machined group) or RBM-treated (48 OMIs, RBM group). RBM treatment was performed with CaP and HNO_3_ (Figure 1) by the OMI product company. Mechanical study at 2 weeks and 4 weeks and histological study were done each with 16 OMIs of machined group and 16 OMIs of RBM group.

### 2.2. Scanning Electron Microscopy, Scanning Interferometry, and Energy-Dispersive Spectrometry

A topographic evaluation was performed using scanning electron microscopy (JSM-840A, JEOL, Tokyo, Japan) to compare the surface structures between the two groups.

Two samples from each group were randomly selected and scanned by the Optical Profiler (Wyko NT8000, Veeco, Tucson, AZ, USA) for analyzing the following surface roughness parameters: *R*_*a*_, which was the arithmetic average deviation in the roughness profile from the mean line; *R*_*q*_, which was the root mean square height corresponding to *R*_*a*_; and *R*_*z*_, which was the maximum peak to valley height in the evaluated area.

### 2.3. Animals

This study was approved by the Ethics Committee for Research on Animals (Seoul National University Ethical Board, SNU 120308-2). In all, twenty-four 3-month-old New Zealand white rabbits (mean weight, 3.5–4.0 kg) were included; of these, 16 rabbits were used for the mechanical studies and 8 rabbits for the histological studies.

### 2.4. Surgical Procedures

The rabbits were anesthetized with an intravenous injection of tiletamine/zolazepam (Zoletil® 50, Virbac, 7.5 mg/kg) and xylazine 2% (Rompun, Bayer, 0.15 mL/kg). Then, 0.5 mL of 2% lidocaine (1 : 100 000 epinephrine) was applied to the surgical site to provide local anesthesia.

**Figure 2:**
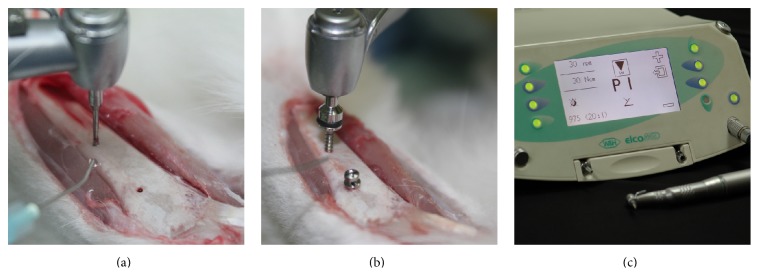
Procedure and equipment for placement of the orthodontic mini-implant. (a) Predrilling into the tibia of rabbits using a surgical implant engine under saline irrigation. (b) Insertion of the mini-implant under 20 rpm with measuring torque. (c) Surgical engine that can measure and record the torque.

One mini-implant from each group was inserted into each tibia of rabbits using a surgical implant engine (Elcomed SA200C, W&H, Bürmoos, Austria) after predrilling (Ø 1.0 mm) under saline irrigation (Figure 2). Postoperatively, ketoprofen (7 mg/kg) as an anti-inflammatory drug and gentamicin (5 mg/kg) as an antibiotic were administered subcutaneously for 1 week from the day of operation.

For one week after surgery the rabbit's body weight was recorded daily. The rabbit's general condition as well as condition of the surgical wound was evaluated every day for as long as needed.

### 2.5. Evaluation of Insertion and Removal Torques and Removal Angular Momentum

**Figure 3:**
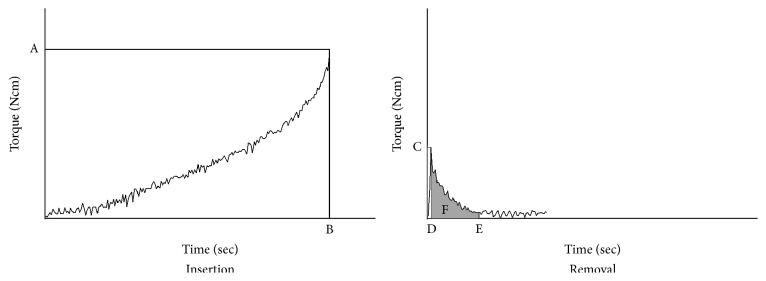
Schematic graph to explain the mechanical characteristics of mini-implants. (A) Maximum insertion torque (MIT). (B) Time of MIT. (C) Maximum removal torque (MRT). (D) Time of MRT. (E) 1.5 seconds after MRT, which is the time needed for a half turn. (F) Removal angular momentum (RAM), which is the torque integrated from the time of MRT to 1.5 seconds under 20 rpm after MRT.

Sixty-four OMIs were inserted and half of them were removed at 2 weeks and another half at 4 weeks after implantation. Torque values were recorded by the surgical engine (Figure 2(c)) [22], which had a rotational speed of 20 rpm. Impdat software (Kea Software GmbH, Poecking, Germany) was used for the readout of the recorded torque value. The torque was recorded 8 times per second. Maximum insertion torque (MIT), maximum removal torque (MRT), and removal angular momentum (RAM) were measured to evaluate the mechanical stability (Figure 3). To analyze the energy required to remove the mini-implant to the bone, the removal angular momentum (RAM, Ncms) was calculated by integrating the torque during a half turn, which took 1.5 seconds under 20 rpm, after MRT.

### 2.6. Fluorescence Bone Labeling

Two fluorochromatic dyes were used for fluorescence bone labeling. Tetracycline hydrogen chloride (Sigma, St. Louis, MO, 15 mg/kg) was intramuscularly injected on the first day after implantation, while calcein (Sigma, St. Louis, MO, 10 mg/kg) was intramuscularly injected at 2 weeks after implantation.

### 2.7. Specimen Preparation

Eight rabbits were euthanized for histomorphometric analysis at 4 weeks after implantation. Mini-implant specimens with the surrounding tissue were embedded in light-curing resin (Technovit 7200VLC; Heraeus Kulzer, Dormagen, Germany), sliced, and ground into 40–50 *μ*m using the Exakt cutting and grinding system (Exakt Apparatebau, Norstedt, Germany) [23]. Specimens were stained with hematoxylin and eosin (HE).

### 2.8. Histomorphometric Analyses

Each specimen of 32 OMIs (16 of machined group and 16 of RBM group) was observed using a fluorescence microscope (Nikon Eclipse TE 200 microscope, Nikon, Tokyo, Japan) before staining. Histological examinations were performed using an Olympus BX51 microscope (Olympus Co., Tokyo, Japan). The following parameters [24] for the 3 best consecutive threads were measured using image analyzing software (KAPPA, Optoelectronics GmbH, Kleines Feld, Germany): the bone-to-implant contact (BIC), which was the percentage of total bone contact length on the threads of the implant, and bone area (BA), which was the percentage of total BA within the threads of the implant.

### 2.9. Statistical Analysis

All measurements were statistically evaluated using the independent *t*-test to determine any difference in MIT, MRT, RAM, BIC, and BA between the machined and RBM groups. A *P* value of <0.05 was considered statistically significant.

## 3. Results

### 3.1. Topographic Findings

**Figure 4:**
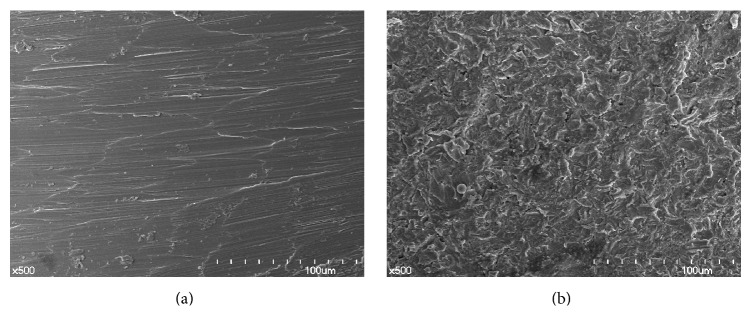
Scanning electron microscopy findings. (a) Machined group. (b) Resorbable blasting media (RBM) group. Although the surface of the machined group showed smooth surfaces with linear scratches, the RBM group showed a rough surface with irregular indentation.

**Figure 5:**
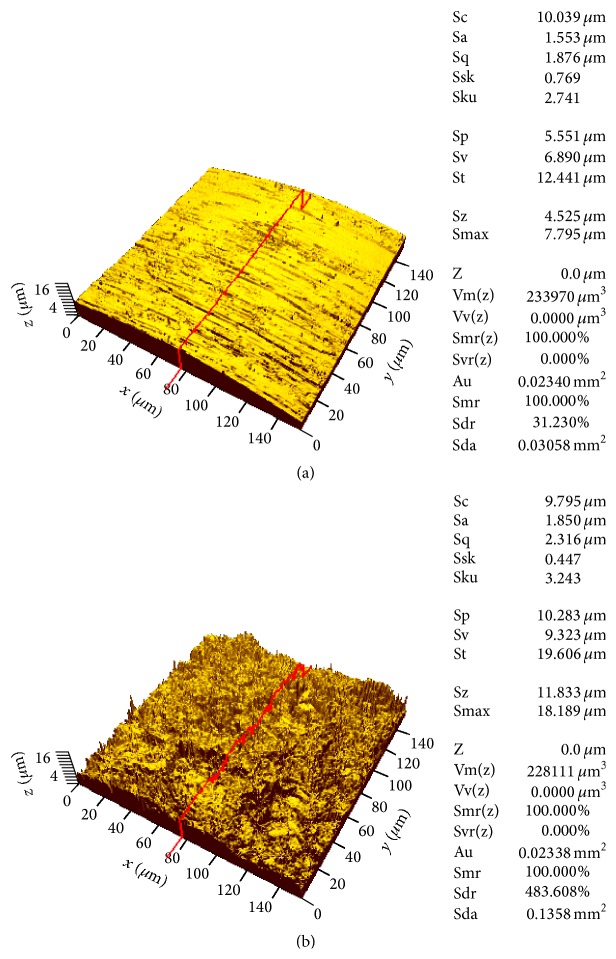
Three-dimensional roughness of the analyzed OMI's surfaces. (a) Machined group. (b) Resorbable blasting media (RBM) group. The surface of the RBM group was rougher than that of the machined group. The peak to valley height in the evaluated area was higher and the roughness surface area was larger in the RBM group than in the machined group.

Scanning electron microscopy analysis demonstrated that the implant surfaces in the RBM group were rough and irregular, while those in the machined group were relatively smooth (Figure 4). The surfaces in the RMB group appeared reticulated with undermining deformation of the remaining metal. Overall, the roughness of implant surfaces was greater in the RBM group (*R*_*a*_, 1.66 *μ*m; *R*_*q*_, 1.94 *μ*m; and *R*_*z*_, 5.02 *μ*m) than in the machined group (*R*_*a*_, 0.51 *μ*m; *R*_*q*_, 0.61 *μ*m; and *R*_*z*_, 1.64 *μ*m) (Table 3 and Figure 5).

### 3.2. Mechanical Assessments

The RBM group exhibited a significantly lower MIT and a significantly higher MRT and RAM compared with the machined group at 2 weeks after implantation (*P* < 0.05, Tables 4, 5, and 6). However, at 4 weeks after implantation, MRT and RAM showed no significant difference between the groups.

### 3.3. Histomorphometric Findings

At 4 weeks after implantation, there was no significant difference in BIC between the machined and RBM groups (Table 7), whereas BA was significantly higher in the RBM group than in the machined group (*P* < 0.05).

### 3.4. Histological Findings

**Figure 6:**
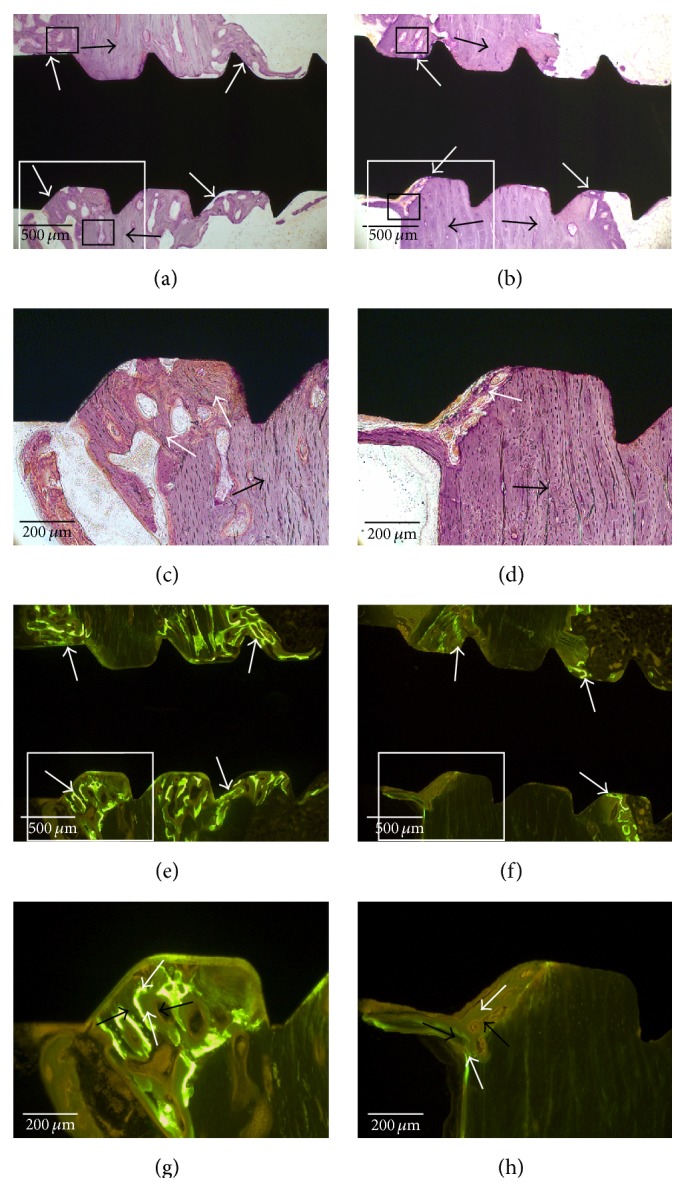
Histological specimens from the machined group (a, c, e, and g) and resorbable blasting media (RBM) group (b, d, f, and h). Microscopic views of hematoxylin-eosin-stained sections are observed from (a) to (d), and fluorescence microscopic views are observed from (e) through (h). (a) In the machined group, a large area of new bone formation (white arrows) surrounds the resorbed old bone (black arrows; ×4). (b) In the RBM group, there is a large area of intact old lamellar bone (black arrows) and little new bone formation (white arrows; ×4). (c) A magnified view of the white box in (a). New bone (white arrows) occupies the area of the resorbed old bone (black arrow; ×10). (d) A magnified view of the white box in (b). Old lamellar bone (black arrow) is resorbed with a thin surface margin and little new bone formation (white arrow; ×10). (e) Fluorescence bone labeling image of (a). Green color (calcein, white arrows) is widely observed at the bone-mini-implant interface (×4). (f) Fluorescence bone labeling image of (b). There is a small green-colored area (calcein, white arrows; ×4). (g) A magnified view of the white box in (e). A green-colored area (calcein, upper white arrow) which was formed at 2 weeks after mini-implant insertion is observed beside old bone (left black arrow). The other new bone (lower white arrow) which was formed after 2 weeks is observed between upper white arrow and left black arrow. There are scattered large green color areas. (h) A magnified view of the white box in (f). Thin green lines can be observed (calcein, lower white arrow) around remaining old bone (left black arrow). There is an area which was formed after 2 weeks between green line (lower white arrow) and resorbed area (right black arrow). The new formed bone is smaller than new bone of (g).

**Figure 7:**
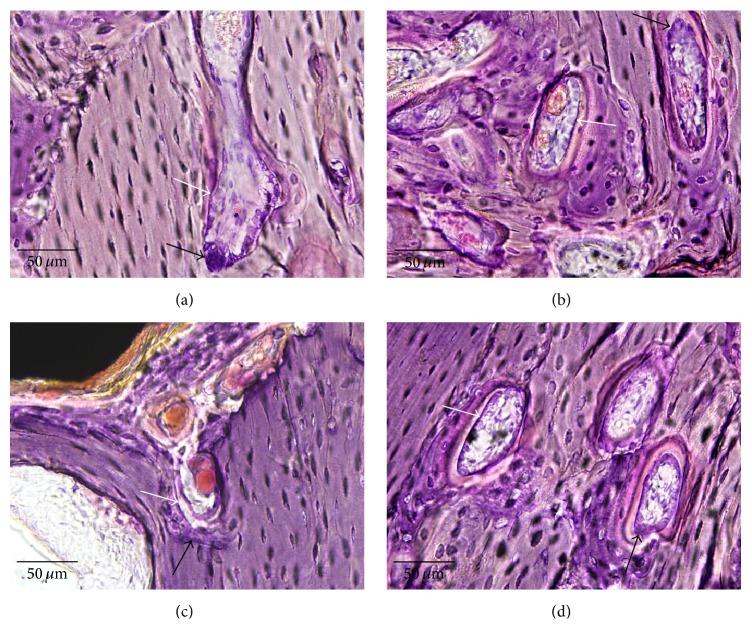
Higher magnification histology images of machined group ((a) and (b), black boxes in Figure 6(a), ×40) and RBM group ((c) and (d), black boxes in Figure 6(b), ×40). (a) An active bone cone with osteoclasts (black arrow) and osteoblasts (white arrow) is in old lamella bone. (b) There are several active bone cones which have osteoclasts (black arrow) and osteoblasts (white arrow) in old lamella bone and new bone. (c) There are few bone cones which show unclear osteoclasts and osteoblast (black and white arrows). (d) Although there are several bone cones in old lamella bone, they are less active than (b) and they have unclear osteoclast (black arrow) and osteoblast (white arrow).

Results of light microscopy revealed new bone formation after old bone resorption around the OMI surfaces. New bone growth was observed on the OMI surfaces on the marrow side (Figures 6(a) and 6(c)). The old lamella bone was more evident in the RBM group than in the machined group (Figures 6(b) and 6(d)). However, bone remodeling was greater in the machined group than in the RBM group. Fluorescence microscopy revealed only calcein deposition, which was green, compared with tetracycline deposition, which was orange or yellow, in both the groups (Figure 6(e)). In machined group, active osteogenic cells such as osteoblast and osteoclast were found to be more than RBM group (Figure 7).

## 4. Discussion

In topographic analysis, *R*_*a*_ in the RBM group was 1.66 *μ*m, which was higher than that in the machined group and close to the optimal surface roughness (1.0–2.0 *μ*m) for adequate retention in the bone [25]. Therefore, RBM-treated implants might be associated with initial bone formation on the implant surface in contact with bone without extensive bone resorption in the early stage after implantation, thus maintaining early stability.

The histological and fluorescence results of this study showed that the machined group had broad old bone resorption and new bone apposition at 2 weeks after implantation. There was little tetracycline, which was injected 1 day after implantation. This finding may suggest that there is more bone resorption than bone apposition during the early stage after implantation. However, less bone resorption and more old lamellar bone were observed in RBM group. This suggests that the RBM surface could prohibit initial bone resorption and maintain the stability of OMI after implantation; in contrast, the machined surface could induce bone resorption and new bone remodeling, resulting in low stability in the early stage.

Histomorphometric analysis revealed similar BIC in both groups at 4 weeks after the implantation (Table 7). However, BA was lower in the machined group than in the RBM group because there was more old bone resorption and new bone apposition in the former than in the latter. This phenomenon may decrease the early stability because the formation of new bone and its transition into old bone may require some time. Another study [26] reported similar results that OMIs placed in the mandible show decreased stability during the first 3 weeks and increased stability over the next 2 weeks.

Therefore, the lower MRT at 2 weeks after implantation in the machined group compared with that in the RBM group may be attributed to the greater initial old bone resorption in the former during the 2 weeks after implantation. After old bone resorption, there was greater new bone apposition in the machined group than in the RBM group, and MRT in the machined group recovered at 4 weeks after implantation. Machined group showed more osteogenic cell such as osteoblast and osteoclast then RBM group. The low MIT in the RBM group may have been caused by surface blasting treatment, which could have decreased the OMI diameter and the decreased diameter of OMI could have affected the MIT of the RBM group [27]. Therefore the null hypothesis, which stated that RBM surface treatment would not significantly affect the initial stability of OMIs, was rejected. Although the stability of implants in the machined group may be decreased during the first 2 weeks after implantation, it may recover by 4 weeks after the procedure [28].

The results of this study suggest that RBM surface treatment can preserve the intact old lamellar bone in the early stages after implantation, indicating that RBM surface treatment can support the early stability of OMIs and that OMIs with machined surfaces can have decreased early stability because of greater surrounding bone resorption in the early stage, although this stability can recover through new bone apposition after old bone resorption.

## 5. Conclusions

RBM surface treatment can support the early stability of OMIs, while stability of machine treatment can decrease in the early stage after implantation because of greater surrounding bone resorption. However, the stability of OMIs with machined surfaces can recover through new bone apposition after old bone resorption.

## Figures and Tables

**Table 1:** Chemical composition and mechanical properties of Ti-6Al-4V alloy.

Alloy	Chemical composition (%)								Mechanical properties		
	N	C	H	Fe	O	Al	V	Ti	Tensile strength (MPa)	Young's modulus (GPa)	Yield strength, 0.2% offset (MPa)
Ti-6Al-4V	0.05	0.08	0.012	0.25	0.13	5.5–6.5	3.5–4.5	Balance	860–896	110	795–827

**Table 2:** Number of OMIs used in each study.

	Number of OMIs		
	Machined group	RBM group	Total
Mechanical study			
Removal at 2 weeks after insertion	16	16	32
Removal at 4 weeks after insertion	16	16	32
Histological study			
Specimen preparation at 4 weeks after insertion	16	16	32

Total	48	48	96

**Table 3:** Surface roughness of machined group and RBM group in topographic evaluation.

	Surface roughness (*μ*m)	
	Machined group	RBM group
*R*_*a*_	0.51	1.66
*R*_*q*_	0.61	1.94
*R*_*z*_	1.64	5.02

*R*_*a*_: arithmetic mean of the departures of the roughness profile from the mean line.

*R*_*q*_: root mean square parameter corresponding to *R*_*a*_.

*R*_*z*_: maximum peak to valley height in the evaluation area.

**Table 4:** Maximum insertion torque of machined group and RBM group.

Maximum insertion torque (Ncm)				*P* value^*∗*^
Machined group		RBM group		
Mean	SD	Mean	SD	
11.11	2.05	9.57	1.47	0.001^†^

SD: standard deviation.

^*∗*^Independent *t*-test; ^†^ *P* < 0.01.

**Table 5:** Maximum removal torque of machined group and RBM group at 2 weeks and 4 weeks after the implantation.

Time	Maximum removal torque (Ncm)				*P* value^*∗*^
	Machined group		RBM group		
	Mean	SD	Mean	SD	
2 weeks	5.45	1.35	7.06	1.78	0.007^†^
4 weeks	6.41	2.3	7.08	3.12	0.530

SD: standard deviation.

^*∗*^Independent *t*-test; ^†^ *P* < 0.05.

**Table 6:** Removal angular momentum of machined group and RBM group at 2 weeks and 4 weeks after the implantation.

Time	Removal angular momentum (Ncms)				*P* value^*∗*^
	Machined group		RBM group		
	Mean	SD	Mean	SD	
2 weeks	6.45	1.67	7.9	1.86	0.026^†^
4 weeks	13.62	5.07	12.52	2.93	0.489

Removal angular momentum was the integrated removal torque during the first half turn.

SD: standard deviation.

^*∗*^Independent *t*-test; ^†^ *P* < 0.05.

**Table 7:** Histomorphometric analysis of the machined group and RBM group in noncalcification specimens at 4 weeks after the implantation.

Measurement	Machined group		RBM group		*P* value^*∗*^
	Mean	SD	Mean	SD	
BIC (%)	68.21	9.12	69.23	12.53	0.793
BA (%)	67.99	10.68	77.30	11.82	0.026^†^

Bone-to-implant contact (BIC) and bone area (BA) were measured.

SD: standard deviation.

^*∗*^Independent *t*-test; ^†^ *P* < 0.05.
